# A randomised crossover trial of five cryocompression devices’ ability to reduce skin temperature of the knee

**DOI:** 10.1371/journal.pone.0296634

**Published:** 2024-01-16

**Authors:** James Belsey, Andrew Reid, Eloise Paine, James Faulkner

**Affiliations:** School of Sport, Health & Community, Faculty of Health & Wellbeing, University of Winchester, Winchester, Hampshire, United Kingdom; IRCCS Istituto Ortopedico Rizzoli, ITALY

## Abstract

**Background:**

The application of cold and pressure to the knee is a common part of post-operative rehabilitation. Skin temperature should be reduced to within 10–15 °C to optimise the therapeutic benefits of cryocompression. The purpose of this study was to investigate the ability of five different cryocompression devices to reduce skin temperature to within this therapeutic range.

**Materials and methods:**

32 healthy adult participants (mean (SD): age 26.3 (7.9) years; BMI 24.8 (2.7) kg/m^2^; 20 males) were recruited for this randomised crossover study. Skin temperature was measured 20 mm distal to the patella using a k-type thermocouple every five minutes during a 30-minute treatment with one of five different cryocompression devices (Physiolab S1, GameReady, Cryo/Cuff, VPulse, and a Gel Wrap). Changes in skin temperature over time were compared to baseline within and between conditions. A subjective rating of comfort was also recorded for each device.

**Results:**

The Physiolab S1 and GameReady devices caused significantly lower skin temperatures compared to the VPulse, Gel Wrap, and Cryo/Cuff after 30 minutes (p<0.05). 87–96% reported a positive comfort rating for the Physiolab S1, VPulse, Cryo/Cuff and Gel Wrap, whereas 53% of participants reported a positive comfort rating for the GameReady.

**Conclusions:**

Only the Physiolab S1 and GameReady devices reduced skin temperature of the knee to within the target range of 10–15 °C. The Physiolab S1 was reportedly more comfortable than the GameReady. Clinicians should be aware of the performance differences of different cryocompression devices to understand which is most likely to provide an effective dose of cold therapy to a joint.

## Introduction

The application of cold and pressure to a joint after surgery is known to reduce pain and swelling, while improving range-of-motion and patient satisfaction with their outcome [1–3]. Cold application is intended to reduce intra-muscular and intra-articular temperature to a degree that attenuates metabolic activity, causes vasoconstriction, and raises pain thresholds, thereby reducing inflammation, blood loss, and pain [4, 5]. Simultaneous compression compliments cold application by supporting vasoconstriction and aiding venous return, which further combats pain and swelling [6, 7].

Reducing absolute skin temperature, via a cryocompression modality, to within 10–15 °C has been shown to correspond to a reduction in intra-muscular and intra-articular temperature that is sufficient to confer a therapeutic benefit to the recipient of the treatment [8–11]. Absolute skin temperatures >15 °C are sub-optimal for eliciting improvements in clinical outcomes [8, 10, 11], whereas absolute skin temperatures <10 °C increase the risk of adverse reactions to the cold [8, 12, 13].

A number of devices are available for use in post-operative settings that provide patients with a combination of cold and compression therapy. Several clinical studies have been conducted to assess the effect of these devices on surgical outcome, with some demonstrating favourable results when compared to traditional methods (ice packs and bandages) [6, 14–18]. However, inconsistencies emerge when comparing post-operative outcomes between different cryocompression devices [5, 11, 19, 20], indicating a potential variation between devices to provide effective treatments. These differences in reported outcomes when using different cryocompression devices may be suggestive of ineffective cooling; defined as absolute skin temperature failing to drop below 15 °C during a treatment.

To date, there is very little published data relating to the various cryocompression modalities on the market. Depending on the device, this leaves clinicians and patients with little-to-no evidence to help inform decisions as to which modality is most appropriate for their needs. To our knowledge, the basic functionality of different cryocompression devices and their ability to successfully reduce skin temperature to within the therapeutic range of 10–15 °C has not been previously researched. Developing an understanding around the basic performance capabilities of different devices will provide guidance as to which device, if any, is able to provide an effective treatment to the user. This information can be used by hospitals and clinicians to better help justify decisions that are made when opting to purchase and use a particular cryocompression modality.

Therefore, the purpose of this study was to investigate the ability of five different cryocompression devices to reduce skin temperature to within the therapeutic range during a 30-minute treatment, in line with standard guidelines [11, 21] and the manufacturer’s recommendations. It was hypothesised that all devices would significantly reduce skin temperature compared to pre-treatment levels, but that not all devices would successfully reduce skin temperature to within 10–15 °C.

## Materials and methods

### Study design

This study implemented a randomised crossover design (S1 Checklist) with a random number generator being used to determine the test order for each participant. Randomisation was conducted by the lead author (JB), and two of the authors (JB and AR) were responsible for participant enrolment. Five different cryocompression devices corresponded to the five independent conditions completed by each participant. A random number generator was used to determine the leg of each participant that would be used. Once determined, the same leg was used for each test. A minimum of 24 hours was left between tests with the same participant to allow the leg to fully return to a normal temperature before the next treatment. All participant recruitment and testing took part from 15/03/2022 to 05/12/2022 within a laboratory environment and all participants provided written informed consent prior to their involvement in the study. A power analysis was conducted a-priori based on an analysis of variance (ANOVA) with repeated measures, which showed that 30 participants (150 observations) would be required to achieve a power of 0.8 and alpha error of 0.05, for a small-medium effect size of f = 0.2. Ethical approval was granted by the University of Winchester (UK) Faculty of Health and Wellbeing ethics committee on 11/03/2022 (ID: HWB_REC_220228). The study was pre-registered in the ClinicalTrials.gov registry (ID: NCT05355116), and subsequently also registered retrospectively in the ISRCTN registry (ID: ISRCTN51736090) to comply with the PLOS ONE list of acceptable registries. The authors confirm that all ongoing and related trials for this intervention are registered.

### Participants

Volunteers >18 years old at the point of recruitment were considered for participation. Thirty-two healthy adult participants (mean (SD); age 26.3 (7.9) years; BMI 24.8 (2.7) kg/m^2^; 20 males) were recruited from a university population between March and December 2022, and provided written informed consent prior to any data being collected. Participants were initially screened using a self-report questionnaire prior to testing to avoid including anyone who would be normally contraindicated for cryocompression therapy (Table 1) [11, 14, 22]. Participants each underwent five tests: one within each of the five study conditions (S1 Protocol).

**Table 1:** Exclusion criteria.

• Body mass index >40 kg/m^2^
• History of nerve damage or sensory deficit in the lower limbs (inc. frostbite)
• Hypersensitivity to cold (inc. hives)
• Active inflammation or pain of the knee
• History of thrombosis, embolism, or other conditions related to impaired peripheral circulation
• Suffering from diagnosed diabetes, multiple sclerosis, rheumatoid arthritis, spinal cord injury, cardiovascular disease, hypertension, Raynaud’s phenomenon, cryoglobulinemia, or hemoglobinuria
• Confirmed or suspected tissue infection, an unstable fracture, a skin condition, or tumour in the treatment area
• Cognitive impairment or communication barriers

### Procedures

The five cryocompression devices for the knee used in the study–which constituted the five conditions–were: Physiolab S1 (Physiolab Technologies Ltd, Milton Keynes, UK), Breg VPulse (Breg Inc, Carlsbad, California, USA), Aircast Cryo/Cuff (DJO Global, Vista, California, USA), GameReady (CoolSystems Inc, Concord, California, USA), and a Gel Wrap (Koolcare Technology Ltd, Shanghai, China). Each device applies a combination of cold and compression during treatments. Three of the devices (Physiolab S1, VPulse, and GameReady) are controlled electronically to automatically provide a user-controlled continuous cold-flow of water and dynamic intermittent pneumatic pressure throughout treatments. They each consist of a cuff that spans from the mid-calf to mid-thigh, connected to the electronic cooling unit via a tube. The Cryo/Cuff is a manual system, which uses gravity to fill and empty the cuff as required in order to ensure a continuous application of cold to the knee. The temperature applied by the Cryo/Cuff is non-modifiable. The static pressure applied by the Cryo/Cuff is variable and depends upon the length of time and height from which the cold water is dispensed from the cooler, through a tube, to the cuff. The manufacturer recommends that the Cryo/Cuff is filled for no more than 30 seconds from a height of no more than 15 inches (38 cm) to avoid the occurrence of unwanted adverse effects due to the application of extreme hydrostatic pressure. The Cryo/Cuff is then drained and refilled with cold water during application, as required, when the water inside the cuff begins to re-warm. Finally, the Gel Wrap uses three gel packs which are first placed in a freezer for at least two hours before being inserted into the wrap and applied to the knee. The temperature and static pressure applied by the Gel Wrap are not controllable and there is no continuous cold-flow during application.

Participants were positioned in a seated position on a therapy bed with the head of the bed in its maximum upright position, and with legs elevated and in full extension, parallel to the floor for the duration of each test. A k-type thermocouple was then taped 20 mm distal to the patella in order for skin temperature to be measured during testing. This location was selected for skin temperature measurement as it was central within the treatment area but not directly over the patella, which has previously been noted to act as a heat shield [10], which could produce misleading results. An acclimatisation period then ensued with skin temperature being measured every minute for 5 minutes or until the temperature readings where consistent to within 0.5 °C for three consecutive minutes; which was longest.

Following the acclimatisation period, and depending on the randomised test order, one of the five cryocompression devices was applied to the leg of participant according to the manufacturer’s instructions, with the knee positioned centrally within the cuff. The test then began by setting the device to the manufacturer’s recommended guidelines. Table 2 gives an overview of the temperature and pressure settings used with each device.

**Table 2:** Manufacturer’s recommended settings for the five cryocompression devices used in the study.

Device	Temperature (°C)	Pressure (mmHg)	Type of pressure
Physiolab S1	8	25–50	Dynamic intermittent
VPulse	5.5	Unknown	Dynamic intermittent
Cryo/Cuff	Unknown	~30	Static
GameReady	1	5–50	Dynamic intermittent
Gel Wrap	Unknown	Unknown	Static

Skin temperature was measured prior to the application of the cryocompression device to the leg, and then at single time-points every 5 minutes during a treatment. The device was applied for 30-minutes for each test before the cuff was removed from the leg. If skin temperature was within 10–15°C at the end of a 30-minute test, it would continue to be monitored every 5 minutes until it reached >15°C. This allowed for the total time spent with skin temperature within the target therapeutic zone of 10–15°C to be measured. In tests where skin temperature did not dip below the 15 °C threshold, the test was deemed complete and the cuff was removed from the leg.

The primary outcomes were changes in skin temperature over time compared to baseline values within each condition; and differences in skin temperature over time between conditions. Secondary outcomes included the time taken to achieve a skin temperature of 10–15°C, and the time spent within this range. The mean difference between device temperature settings and measured skin temperature settings at the end of a test was also measured. Finally, a subjective rating of comfort was also recorded using a Likert scale. Participants were asked “How comfortable did you find the treatment you just experienced?”, and were asked to provide one of the following answers: very comfortable, comfortable, neutral, uncomfortable, or very uncomfortable.

### Statistical analysis

A Kolmogorov-Smirnov test was first conducted to assess the normality of the data. An ANOVA with repeated measures was performed to assess skin temperature changes over time within and between conditions. Where relevant, a post-hoc correction was conducted using Tukey’s HSD test to determine any significant differences within and between conditions over time. Independent samples t-tests were performed to detect any differences in skin temperature between the categorical demographic variables of sex and leg. Finally, Pearson correlations were calculated to identify any relationships between skin temperature and the demographic variables of age, height, mass, and body mass index (BMI). All statistical analysis was performed using IBM SPSS Statistics 27 software and differences were considered significant if p<0.05.

The analysis of the subjective ratings of comfort was conducted by considering responses to be “Positive” when treatments were either deemed “Very comfortable” or “Comfortable”, and “Negative” when treatments were deemed either “Uncomfortable” or “Very uncomfortable” by participants.

## Results

### Baseline measures

**Fig 1:**
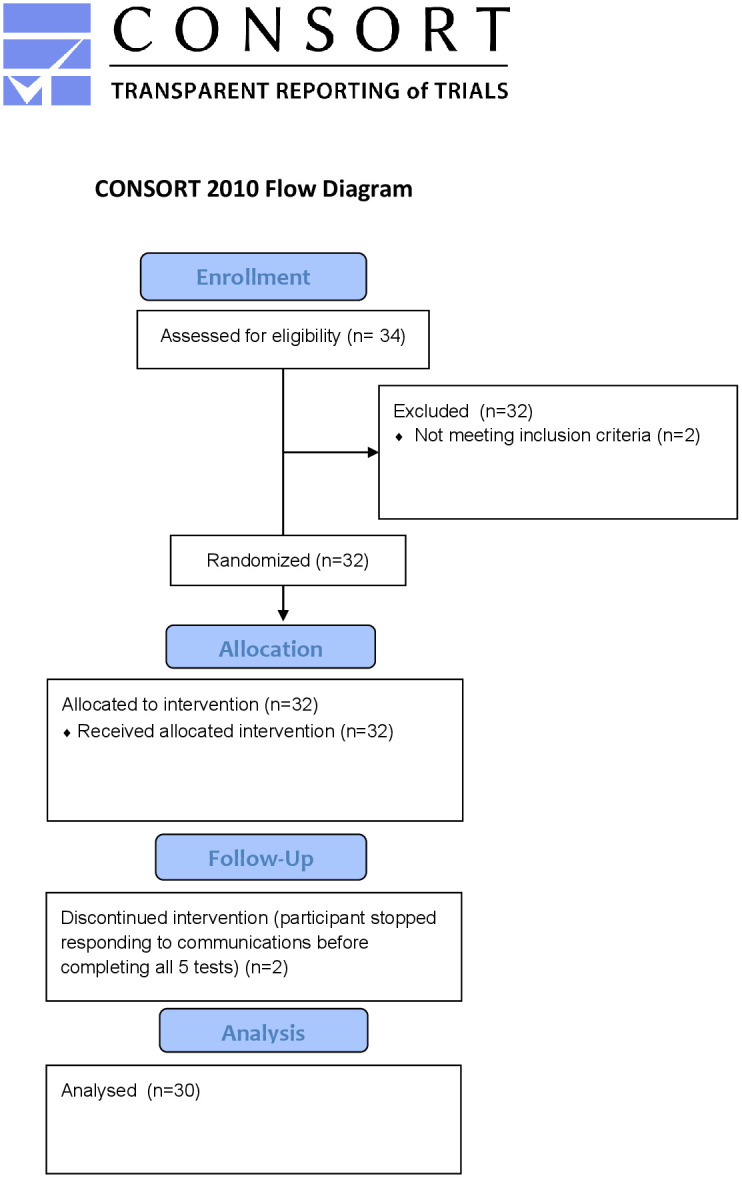
CONSORT flow diagram of participant recruitment process.

Thirty-two participants were originally recruited between March and December 2022 but two withdrew for unknown reasons and did not complete all five tests (Fig 1). Therefore, the results presented are based on the remaining 30 participants (S1 Dataset). Full demographic information of these participants can be found in Table 3.

**Table 3:** Participant demographics.

Characteristic	*n* or mean (SD)
Participants (n)	30
Mean age (years)	26.6 (8.0)
Sex (M:F)	18:12
Leg (L:R)	15:15
Mean height (m)	1.75 (0.1)
Mean mass (kg)	76.2 (10.4)
Mean BMI (kg/m^2^)	24.8 (2.8)

The mean ambient temperature for each test was 21.8 (1.1) °C with no significant differences between conditions. The mean baseline skin temperature for each test was 31.4 (1.8) °C with no significant differences between conditions.

### Primary outcome measures

**Fig 2:**
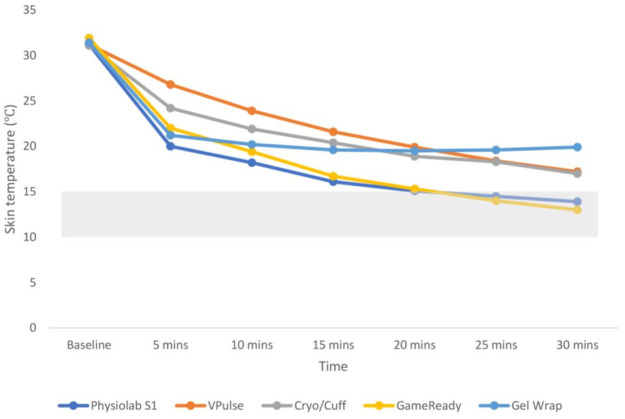
Mean skin temperature during a 30-minute treatment using five different cryocompression devices. Shaded area indicates the target therapeutic zone of 10–15 °C.

Fig 2 shows the mean skin temperature at each measurement point during a 30-minute treatment with each cryocompression device. A significant condition-by-time interaction was observed (p<0.001, *η*^2^ = 0.48). The Physiolab S1 and GameReady caused significantly lower skin temperatures compared to the VPulse and Cryo/Cuff at all time points during the 30-minute treatment (p<0.05). They also caused significantly lower skin temperatures compared to the Gel Wrap between 15 and 30 minutes (p<0.01). No significant differences in skin temperature were detected between the Physiolab S1 and the GameReady at any time point. The Gel Wrap achieved significantly lower skin temperatures compared to the VPulse after 5, 10 and 15 minutes, and reached significantly higher skin temperatures compared to all other devices after 30 minutes. The Cryo/Cuff achieved significantly lower skin temperatures compared to the VPulse, but significantly higher skin temperatures compared to all other devices after 5 minutes. The VPulse achieved significantly higher skin temperatures compared to all other devices after 5 minutes.

The Physiolab S1, VPulse and GameReady reduced skin temperature significantly at each time point for the first fifteen minutes of the treatment to 16.1 (1.2) °C, 21.6 (2.9) °C, and 16.7 (2.6) °C, respectively, at which point temperature reductions continued but not to a statistically significant degree. The Cryo/Cuff reduced skin temperature significantly for the first five minutes of the treatment to 24.2 (3.2) °C, at which point temperature reduction continued but not to a statistically significant degree. The Gel Wrap reduced skin temperature significantly for the first five minutes of the treatment to 21.2 (3.2) °C, at which point skin temperature reductions continued until 20 minutes into the treatment, but not to a statistically significant degree. After 20 minutes with the Gel Wrap, mean skin temperatures began to increase, but not to a statistically significant degree (Fig 2). Only the Physiolab S1 and GameReady were within the target therapeutic range at 30 minutes with mean (SD) skin temperatures of 13.9 (0.8) °C and 13.0 (1.6) °C, respectively. Mean skin temperatures for the VPulse, Cryo/Cuff, and Gel Wrap were 17.2 (2.6) °C, 17.0 (2.9) °C, and 19.9 (2.8) °C, respectively.

Age, sex, and leg were found to have no significant influence on skin temperature during cryocompression treatments (p>0.05) with their related correlation coefficients indicated no-to-weak correlation. As demonstrated in Table 4 significant low-moderate correlations were detected between skin temperature and height, mass, and BMI for the Physiolab S1, Cryo/Cuff, and Gel Wrap at various timepoints. There were no significant correlations between skin temperature and height, mass, and BMI with the VPulse and GameReady (p>0.05) with related correlation coefficients indicating no-to-weak correlations.

**Table 4:** Correlations between skin temperature and height, mass, and BMI (Pearson’s *r*).

	Baseline	5 mins	10 mins	15 mins	20 mins	25 mins	30 mins
**Height (m** **)**							
*Cryo/Cuff*	**-0.39** *	-0.22	**-0.46** *	**-0.40** *	-0.30	**-0.38** *	**-0.39** *
**Mass (kg)**							
* Cryo/Cuff*	0.04	-0.10	-0.24	-0.33	-0.30	**-0.41** *	-0.35
* Gel Wrap*	-0.03	0.12	0.21	0.30	0.33	**0.36** *	**0.36** *
**BMI (kg/m** ^ **2** ^ **)**							
* Gel Wrap*	0.21	0.20	0.27	0.31	0.34	**0.39** *	**0.39** *
* Physiolab S1*	0.07	**0.40** *	0.30	0.30	0.28	0.32	0.32

*significant low-moderate correlation (p<0.05); BMI = Body Mass Index

### Secondary outcome measures

**Fig 3:**
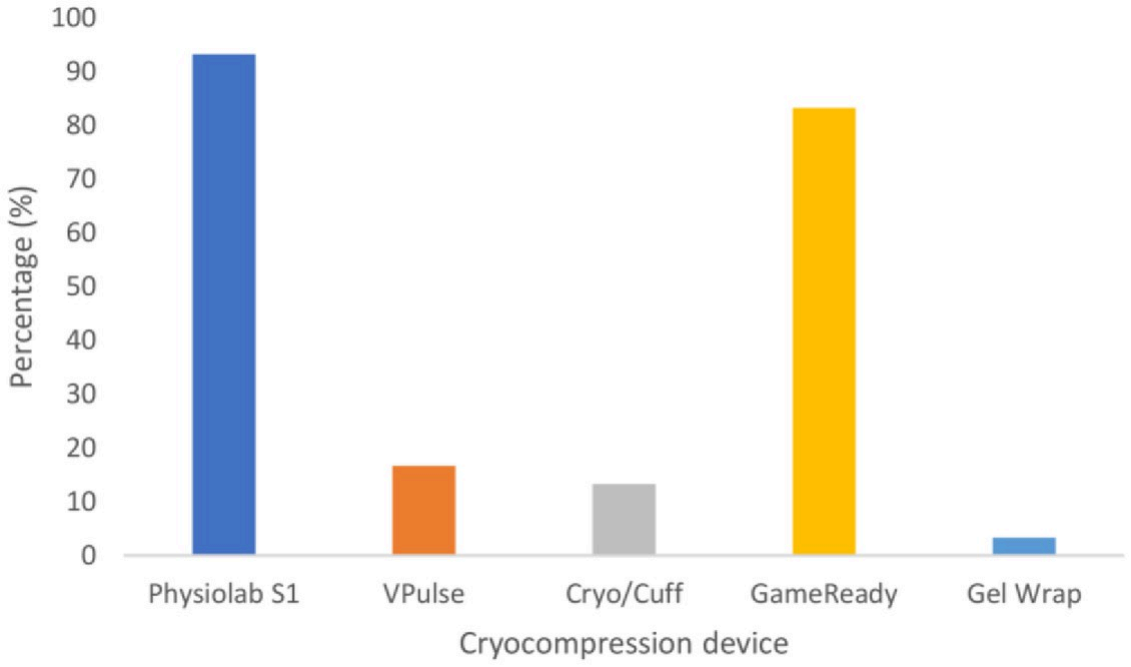
Percentage of participants who achieved skin temperatures within the target therapeutic zone of 10–15 °C within a 30-minute cryocompression treatment.

It took 25 minutes for the Physiolab S1 and GameReady devices to reduce skin temperature to within the target therapeutic range of 10–15°C. 93.3% of participants (n = 28) achieved a skin temperature within the target therapeutic range when using the Physiolab S1 compared to 83.3% (GameReady; n = 25), 16.7% (VPulse; n = 5), 13.3% (Cryo/Cuff; n = 4), and 3.3% (Gel Wrap; n = 1) for the other devices (Fig 3). Of the participants whose skin temperature was within 10–15 °C at the end of the 30-minute treatment, 100% (VPulse, n = 5; Cryo/Cuff, n = 4; and Gel Wrap, n = 1), 92% (GameReady, n = 23), and 89% (Physiolab S1, n = 25) had a skin temperature >15 °C five minutes after the cuff was removed. The remaining participants had a skin temperature >15 °C ten minutes after the cuff was removed, following a cryocompression treatment.

The mean differences between the lowest skin temperature achieved during a 30-minute treatment and the device temperature setting of the Physiolab S1, VPulse, and GameReady was 5.9 (0.8) °C, 11.7 (2.6) °C, and 12.0 (1.6) °C, respectively (p<0.001). It was not possible to provide a similar result for the Cryo/Cuff and Gel Wrap as the temperatures applied by both devices cannot be selected or modified by the user.

**Fig 4:**
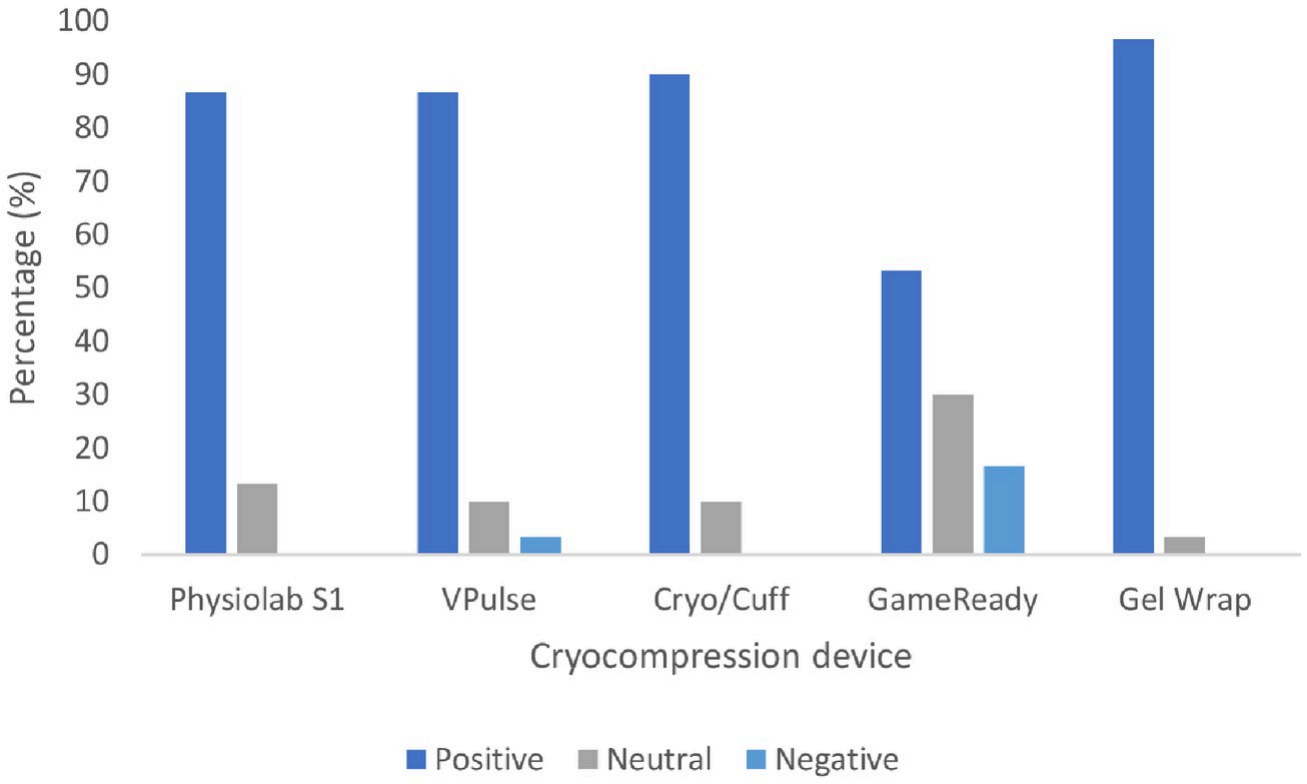
Percentage of participants reporting a positive, neutral, or negative comfort rating after a 30-minute treatment with five different cryocompression devices.

86.7% of participants reported positive comfort ratings for the Physiolab S1 and VPulse compared to 90% (Cryo/Cuff), 53.3% (GameReady), and 96.7% (Gel Wrap) for the other devices. No participants reported negative comfort ratings for the Physiolab S1, Cryo/Cuff, or Gel Wrap. 3.3% of participants for the VPulse and 16.7% of participants for the GameReady reported negative comfort ratings (Fig 4).

## Discussion

The main finding of this study was that the Physiolab S1 and GameReady were the only cryocompression devices that successfully reduced skin temperature to within the therapeutic range of 10–15 °C during a 30-minute treatment, despite all devices reducing skin temperature significantly from baseline. These findings confirmed the hypotheses of the study. Although there were no significant differences in skin temperature between the Physiolab S1 and GameReady, both devices reduced skin temperature significantly more than the VPulse, Cryo/Cuff, and Gel Wrap. The degree to which skin temperature was reduced by the Physiolab S1, Cryo/Cuff, and Gel Wrap was moderately correlated with the height, mass, or BMI of the user. Four of the five devices (Physiolab S1, VPulse, Cryo/Cuff, Gel Wrap) had a positive rating of subjective comfort >86% whereas the GameReady had the lowest positive comfort rating (53.3%) and the highest negative comfort rating (16.7%). Based on these findings, the Physiolab S1 may be considered the preferred device in terms of overall performance and subjective comfort.

According to White and Wells [13], skin temperature reduces most rapidly in the first few minutes of cold exposure, which was confirmed by the results presented in Fig 2. Previous research has demonstrated that cryocompression therapy should reduce skin temperature around the knee to within 10–15 °C in order for the associated therapeutic benefits to be realised [8–11] Absolute skin temperatures within this range are thought to slow sensory nerve conduction velocity and to induce vasoconstriction, which provide an analgesic effect and reduce swelling and blood loss, respectively [23]. The present study demonstrated that two of the five devices under investigation (Physiolab S1 and GameReady) were successful in reducing skin temperature to within this range, suggesting an ability to provide effective cryocompression therapy. However, in both instances, skin temperature only reached the target therapeutic range 25 minutes after the treatment had begun; meaning that the majority of the treatment merely served the purpose of reducing skin temperature in the direction of the target range.

There is some suggestion that intermittent pneumatic compression results in more favourable outcomes during cryocompression therapy [24, 25]. Three of the devices included in the present study (GameReady, Physiolab S1, and VPulse) provided intermittent pneumatic compression to the leg, but only two of these provided an effective treatment in terms of skin temperature reduction. The amount of applied compression between the effective devices (GameReady and Physiolab S1) was similar (up to 50 mmHg), which was greater than that applied by the Cryo/Cuff (circa 30 mmHg). While the compression applied by the VPulse and Gel Wrap was unknown, the observed difference between the Cryo/Cuff and the two effective devices may be an important one as a negative correlation between skin temperature and the amount of applied compression has previously been reported [26]. Further research is needed to confirm the influence of compression on skin temperature reduction, and therefore the level of compression required to optimise the outcome of cryocompression therapy.

Historic guidelines for the application of cold and pressure suggest an optimal duration of 20–30 minutes per treatment [11, 21], which matches the treatment duration in the present study. However, as discussed above, the treatment duration did not equate to the amount of time a user may receive a benefit from the therapy. Methods for reducing skin temperature more quickly within a 30-minute treatment–or seeking to increase treatment duration–in order to optimise the length of time spent with skin temperature within the target therapeutic range, should be investigated.

Once the cuff was removed from the leg following a treatment, absolute skin temperatures rose to above 15 °C after 5 minutes for 89% of participants using the Physiolab S1, and in 92% of participants using the GameReady. It is understood that the application of cold to the skin causes reductions in temperature within the intra-articular space through the removal of heat from warmer deep tissues to colder superficial tissues along a thermal gradient [27]. The rate at which continued cooling occurs within the joint following cold therapy is dependent on the size of the thermal gradient between the deep tissues and the skin [13, 27]. The largest reduction in intra-articular temperature would have been experienced while cold was being actively applied to the skin, but the lowest absolute intra-articular temperature would have been reached during the post-cooling period as temperatures continued to drop further, albeit at a slower rate while the skin gradually re-warmed [13, 27]. This highlights the importance of skin temperature not being reduced below the safe lower threshold of 10 °C during a cryocompression treatment, as the risk of cold injury increases below this mark [8, 12, 13].

Although intra-articular temperatures were not measured in the present study, this known physiological response to cold application indicates that the deeper tissues within the joint most likely continued to cool even further after the removal of the Physiolab S1 and GameReady devices, and after skin temperatures rose above 15 °C. The precise length of time within the post-cooling period that it takes for the intra-articular space to reach its lowest point requires investigation and would help to determine the optimal duration of cryocompression treatments with the Phsyiolab S1 and GameReady devices.

The results in the present study showed that the GameReady performed significantly worse compared to the other devices with regard to subjective comfort ratings. While these comfort ratings were provided by a healthy population, and may therefore differ from those perceived by a patient population with active swelling and pain around the joint, our results do appear to concur with previous research within clinical settings. Alfuth *et al*. (2016) noted that device temperatures above 4 °C are perceived as comfortable during treatments [28]. The GameReady was the only device in the present study set to a temperature below this threshold (1 °C), which is consistent with the lowest percentage of participants who rated the treatment positively (53.3%). 16.7% of participants rated the GameReady device as either “uncomfortable” or “very uncomfortable”, which is lower than a study by Leegwater *et al*., (2017), who recorded discomfort in 28% of participants [29]. As there is no additional research that has explored subjective ratings of comfort for the other devices beyond our findings, further research is needed to confirm the extent to which the comfort ratings recorded in the present study are related to those experienced with each device within patient populations.

The Physiolab S1 and GameReady can both be recommended for use over the other devices in terms of effectively reducing skin temperature during a treatment, however previous research has suggested a link between comfort and compliance with treatment protocols for the application of cold and pressure [16, 30, 31]. The low comfort rating for the GameReady in the present study may therefore lead to higher rates of the non-compliance by users, compared to the other devices investigated. Furthermore, the standard deviation for the final skin temperature measured after a 30-minute treatment was twice as large for the GameReady (1.6 °C) compared to the Physiolab S1 (0.8 °C), suggesting that the latter performs more consistently between users.

A significant discrepancy between the temperature setting of the device and the mean final skin temperature after a treatment was observed with the Physiolab S1, GameReady, and VPulse. This phenomenon has also been demonstrated in previous research using different devices [11, 32]. The mean difference between device temperature setting and skin temperature was around twice as large for the GameReady (12.0 °C) and VPulse (11.7 °C) compared to the Physiolab S1 (5.9 °C). This indicates that the Physiolab S1 more effectively removed heat from the skin in comparison to the other devices. It also demonstrates that the temperature setting of a device should not be used as an indicator of the skin temperature that is achieved during a treatment, as equilibrium can not be expected. This is further evidenced by the present data when considering that the Physiolab S1 and GameReady both achieved similar final skin temperatures after a treatment (13.9 °C and 13.0 °C, respectively) despite a large difference in the temperature setting used (8 °C and 1 °C, respectively). In contrast, the mean final skin temperature with the VPulse device (17.2 °C) failed to reach the 10–15 °C target range, despite having a device temperature setting (5.5 °C) between that of the Physiolab S1 and GameReady.

To our knowledge, no research has been published investigating the performance of the Physiolab S1, VPulse, or Gel Wrap either in clinical settings or with healthy populations. A limited number of studies have investigated the clinical performance of the GameReady device in patients following knee surgery. Results consistently showed that the GameReady reduced pain and improved range-of-motion to a similar degree as traditional ice packs [15, 16, 19], while the device out-performed ice pack controls in terms of reduced blood loss, use of analgesics, and improved functional scores and patient satisfaction [15, 16, 19]. The present study demonstrated that the GameReady successfully reduced skin temperature to within the target therapeutic range, therefore the aforementioned positive clinical results are in line with our findings. Since the Physiolab S1 performed similarly to the GameReady in the present study, it is predicted that it could reasonably also impact clinical outcomes after knee surgery in a similar way, though this needs to be confirmed with an appropriately-powered investigation.

Recent systematic literature reviews have included studies that measured the performance of the Cryo/Cuff in post-operative settings, with some showing no significant improvement in outcomes compared to a control condition, and some showing more favourable results for the Cryo/Cuff for parameters such as pain, swelling, and use of analgesia [1, 33]. These conflicting findings indicate that the Cryo/Cuff is capable of conferring a therapeutic benefit, albeit inconsistently between users. The results of the present study support this trend in the literature, since the mean absolute skin temperature of 17 °C (2.9 °C) °C observed during a treatment was outside of the target range but 13% of participants achieved a skin temperature within the 10–15 °C range.

Temperature reduction was moderately correlated with height, mass, and BMI in the present study with the Physiolab S1, Cryo/Cuff, and Gel Wrap. Similar observations have been made in previous publications, with subcutaneous adipose tissue being specifically identified as a key influencing variable [13, 23, 27]. Individuals with a greater skinfold thickness have been shown to require cryotherapy treatment durations of up to six times longer than those with a lower skinfold thickness in order to achieve similar reductions in tissue temperature [34]. This is evidence to suggest that cold therapy protocols should be bespoke and prescribed on a user-to-user basis, and that this could be an explanation for existing controversy in the literature with regard to post-operative clinical outcomes following cryocompression [3, 20, 27].

Electronic continuous cold-flow cryocompression devices like the Physiolab S1 and GameReady have the potential to make bespoke protocols more achievable compared to traditional ice packs due to the modifiable temperature, pressure, and duration settings that can be selected prior to a treatment. Future studies should seek to develop a better understanding regarding how the added control of these user-modifiable settings can influence the effectiveness of a cryocompression treatment by selecting parameters to specifically suit the user.

### Strengths and limitations

The randomised crossover design was a major strength of this study as it removed the issue of potential confounding demographic variables between participants. This is particularly pertinent considering that some moderate correlations between height, mass, and BMI were observed for three of the cryocompression devices. This study was limited by skin temperature only being measured at one location around the knee. The location of different blood vessels, and the density of fat and lean mass, around the knee could result in skin temperature being reduced to varying degrees at different points around the joint. Another limitation was that the cryocompression was applied directly to the skin of healthy participants as opposed to through a bandage or wound dressing of a post-operative population. This reduces the transferability of the findings to clinical settings. However, these results have demonstrated clear differences in the performance of the different devices regarding their ability to reduce skin temperature of the knee, and have highlighted a number of potential issues with the way in which cryocompression devices are currently utilised within rehabilitation protocols.

## Conclusion

The Physiolab S1 and GameReady devices were capable of reducing skin temperature of the knee to within the target range of 10–15 °C, wherein the associated therapeutic benefits of cryocompression are optimally conferred. The VPulse, Cryo/Cuff, and Gel Wrap failed to reach this temperature range. The Physiolab S1 was reported to be markedly more comfortable than the GameReady, which may have implications for user compliance with treatment protocols in clinical and home-based settings. Clinical studies with the Physiolab S1 are needed to confirm the implications of these findings. Research into the optimal duration and amount of compression during treatments with these devices is also warranted.

## Supporting information

S1 Checklist CONSORT checklist. (DOC)

S1 Protocol Trial protocol. (DOCX)

S1 Dataset Anonymised dataset. (XLSX)
