# Supplementary material for: A randomised crossover trial of five cryocompression devices’ ability to reduce skin temperature of the knee
Source: PLoS One. 2024 Jan 16;19(1):e0296634. doi: 10.1371/journal.pone.0296634 (PMC10790989; doi:10.1371/journal.pone.0296634)
Supplement: S1 Protocol — (DOCX) [file pone.0296634.s002.docx]

**Study protocol:** A randomised crossover trial of five cryocompression devices’ ability to reduce skin temperature of the knee.

Introduction

Cryotherapy after surgery is widely utilised and has numerous practical applications for post-operative rehabilitation. Previous research has suggested that during cold therapy, the skin temperature of the knee should be reduced to 10-15°C to maximise the therapeutic benefits of cooling while avoiding the risk of cold injuries such as nerve damage and frostbite (Wilke and Weiner, 2003; Bleakley, McDonough and MacAuley, 2004). However, a recent study noted that where cryocompression devices have previously been used to reduce the skin temperature <10°C, no complications relating to the device have been reported, suggesting that the risk to the user at these lower temperatures is minimal (Bellon *et al.*, 2019). The temperature range at which a cryocompression device should be set in order to achieve a skin temperature within the therapeutic range of 10-15°C is unknown. Furthermore, there is evidence to suggest that the temperature of the device does not equal that to which the skin is reduced (Selfe *et al.*, 2009). Therefore, it is not sufficient to assume that the temperature setting of a cryocompression device accurately reflects skin temperature. Modern cryotherapy devices often consist of some sort of cuff that can be wrapped around the knee, with a connecting tube to a central unit that supplies and circulates ice-water to and from the cuff in order to cool the intended body part. Such devices offer differing levels of control over the temperature of the ice-water as it leaves the central unit, but nothing is known about how this correlates to the skin temperatures that are achieved during a cryotherapy treatment.

The aim of this study is to determine the ability of five different cryocompression.devices to effectively lower the skin temperature of the treatment area to within the therapeutic range.

Methods

*Design*

This prospective randomised controlled trial will recruit healthy adult volunteers from a university population to participate. To avoid the involvement of individuals with unequal relationships, participants will be recruited through the form of advertisements on staff and student noticeboards, and through an email invitation sent to students within the faculty. A power analysis demonstrated that a total of 30 participants would be required to achieve a power of 0.8 and alpha error probability of 0.05 for a small-medium effect size of f=0.2. Each participant will take part in every condition to eliminate the risk of demographic factors confounding the results. Each of the 5 devices being investigated will comprise each of the test conditions. The order in which participants complete the 5 test conditions will be randomised in advance using a computer random number generator. Testing will involve each device being applied and used according to the manufacturers’ instructions. Where a device has the ability to apply different temperatures and pressures to participants, settings will be selected in accordance with manufacturer guidelines and recommendations for use. Each testing session will take around 60 minutes to complete, depending on the treatment application guidelines of each device, with at least 24 hours between tests imvolvimg the same participant. In total, participants will be giving around 5 hours of time across 5 days to complete their participation.

*Inclusion and exclusion criteria*

Participants ≥18 years old will be considered eligible for the study unless they meet any of the following exclusion criteria, which include the common contraindications for cold therapy (Selfe *et al.*, 2009; Fang *et al.*, 2012; Waterman *et al.*, 2012):

- BMI >40 kg/m^2^
- History of nerve damage or sensory deficit in the lower limbs (including frostbite)
- Hypersensitivity to cold, including hives
- Active inflammation or pain of the knee
- History of thrombosis, embolism, or other conditions related to impaired peripheral circulation
- Suffering from diagnosed diabetes, multiple sclerosis, rheumatoid arthritis, spinal cord injury, cardiovascular disease, hypertension, Raynaud disease, cryoglobulinemia, or haemoglobinuria
- Confirmed or suspected tissue infection, an unstable fracture, a skin condition, or a tumour in the treatment area
- Cognitive impairment or communication barriers where adjustments can not be reasonably made

*Protocol*

All eligible participants will first be required to provide informed consent prior to their participation in the study. Once this has been obtained, participants will each attend 5 individual testing sessions, each lasting no longer than 60 minutes. The height and mass of the participants will be measured during the first testing session in order to calculate BMI, and participants’ age and sex will be recorded and included in the later data analysis.

Participants will be required to remain in a seated position with their leg in full extension and elevated, parallel to the floor, for the duration of each test. The leg that will be used for the study will be randomly selected for each participant, and will remain the same for each condition. Skin temperature will be measured using a thermocouple, which will be attached with tape 20 mm distal to the patella. One of the cryocompression devices (Physiolab S1; Physiolab ice pack; GameReady; Cryo/Cuff; or VPulse) will then be attached to the leg of the participant and the test will begin. Depending on the condition to which a participant has been randomly assigned for a given test, a different device will be used. The temperature and pressure settings for each test will be set according to the manufacturers’ recommendations which vary between devices. The lowest temperature setting that will be used throughout the study will be 1℃ (GameReady device) and the highest pressure setting that will be used is 50 mmHg (GameReady and Physiolab S1 devices). None of the temperature or pressure settings used are deemed to be unsafe or have a high risk of adverse reactions, as long as participants are not contraindicated according to the abovementioned exclusion criteria.

The temperature of the skin will be measured using a k-type thermocouple attached to an infrared thermometer immediately prior to the application of a device, and then every 5 minutes for the duration of the test. The length of a test will be 30 minutes, in accordance with the manufacturers’ instructions/recommendations for each device.

Upon completion of a test, the cuff will be removed. If the temperature of the skin has been reduced to within 10-15℃, skin temperature will continue to be monitored every 5 minutes after the cuff has been removed until it rises above 15℃, at which point the test will end. This will allow for the total time to be measured that skin temperature remains within the target therapeutic range as a result of a single application of the cryocompression device. If the skin temperature is not reduced to within 10-15℃ while the cuff is worn, then the test will end after the cuff is initially removed. Upon completion of the test, the temperature sensor will be removed from the leg of the participant; thus completing their participation in the test. Once each participant has taken part in all conditions, they will have completed their participation in the study.

The following measures will be recorded: skin temperature at each time point, minimum skin temperature achieved, time to achieve minimum skin temperature, length of time with skin temperature within therapeutic range of 10-15℃. After each test, participants will also be asked to respond to the following question using a 5-point Likert scale: “How comfortable did you find the treatment you just experienced?” The possible answers to this question will be: “Very comfortable, Comfortable, Neutral, Uncomfortable, Very uncomfortable”. This will provide insight into differences in perception of the treatment, which could be relevant for the likelihood of protocol adherence by patients in clinical settings.

*Analysis*

The data will be analysed to detect any differences during testing compared to baseline measures, and between groups. All data will be first subjected to a Kolmogorov-Smirnov test to assess whether they are normally distributed. A repeated measures analysis of variance will be performed on all normally distributed data. A Friedman test will be performed on any data that are not normally distributed.

*Ethical issues*

There is a low risk of cold injury to participants if their skin temperature is reduced to <10℃ for any prolonged period of time (>60 mins). The coldest temperature setting for this study is 1℃ (GameReady), which will be applied for 30 mins. A recent study conducted at the University of Winchester demonstrated that device temperature settings do not equal the actual skin temperatures achieved, and it is not expected that skin temperatures will be reduced to lower than 6℃ for more than 15 minutes during any test. Therefore, the risk of harm to the participant is deemed to be minimal for this study.

Participants have the right to withdraw themselves and any collected data from the study at any time during, and for the first 7 days after, their participation without having to give a reason. They also have the right to terminate a test session at any time. Testing shall also be terminated as a result of any adverse reactions that emerge. Adverse reactions (e.g. pain) to the testing protocol are not expected due to the low-risk nature of the study, however any that occur shall be recorded and monitored until things return to baseline/normal. In order to monitor any pain/discomfort that might occur, a Numerical Pain Rating Scale (0-10 scale) will be within sight of the participant at all times during testing: a test will be terminated if reported pain/discomfort exceeds 5/10, though none is expected. Any reported pain (or other adverse reactions) shall be stored along with the data collected for that participant. If a test is terminated for a reason that could be mitigated in future, participants will be offered the opportunity to repeat the test another day, should they wish to continue taking part in the study. If a test is terminated due to an adverse reaction that can not be mitigated in future, the participant (and any collected data) will be thanked for their time and withdrawn from the study.

Conflict of interest declaration

James Belsey (JB) receives payment from Physiolab Technologies Ltd as an external research consultant. Physiolab Techbologies Ltd have had no influence on the design of the study. Although the study is being led by JB, who will proceed in his role impartially, the co-authors of this research will assist with the data collection, analysis, and eventual manuscript preparation to ensure that any unethical investigator biases are avoided. In addition to the consultancy fees for JB, Physiolab Technologies Ltd will provide the equipment to be used in this study. The other authors of this work have no conflict of interest to declare.
